# Meta-analysis of incidence rate data in the presence of zero events

**DOI:** 10.1186/s12874-015-0031-0

**Published:** 2015-04-30

**Authors:** Matthew J Spittal, Jane Pirkis, Lyle C Gurrin

**Affiliations:** Melbourne School of Population and Global Health, The University of Melbourne, Victoria, Parkville 3010 Australia

**Keywords:** Meta-analysis, Person-time, Incidence rate ratio, Sparse data

## Abstract

**Background:**

When summary results from studies of counts of events in time contain zeros, the study-specific incidence rate ratio (IRR) and its standard error cannot be calculated because the log of zero is undefined. This poses problems for the widely used inverse-variance method that weights the study-specific IRRs to generate a pooled estimate.

**Methods:**

We conducted a simulation study to compare the inverse-variance method of conducting a meta-analysis (with and without the continuity correction) with alternative methods based on either Poisson regression with fixed interventions effects or Poisson regression with random intervention effects. We manipulated the percentage of zeros in the intervention group (from no zeros to approximately 80 percent zeros), the levels of baseline variability and heterogeneity in the intervention effect, and the number of studies that comprise each meta-analysis. We applied these methods to an example from our own work in suicide prevention and to a recent meta-analysis of the effectiveness of condoms in preventing HIV transmission.

**Results:**

As the percentage of zeros in the data increased, the inverse-variance method of pooling data shows increased bias and reduced coverage. Estimates from Poisson regression with fixed interventions effects also display evidence of bias and poor coverage, due to their inability to account for heterogeneity. Pooled IRRs from Poisson regression with random intervention effects were unaffected by the percentage of zeros in the data or the amount of heterogeneity.

**Conclusion:**

Inverse-variance methods perform poorly when the data contains zeros in either the control or intervention arms. Methods based on Poisson regression with random effect terms for the variance components are very flexible offer substantial improvement.

**Electronic supplementary material:**

The online version of this article (doi:10.1186/s12874-015-0031-0) contains supplementary material, which is available to authorized users.

## Background

Meta-analysis is widely used in medical research to combine information from independent studies to evaluate the effectiveness of an intervention. When the outcome in each independent study is a binary variable, the data can be viewed as a two-by-two contingency table, with each cell corresponding to counts of events (e.g. the number of people with and without disease) in separate groups, for example, participants assigned to treatment and control arms of an intervention study. The pooled effect size typically of interest – a risk difference, relative risk or odds ratio – is then based on the summary information collected from these studies. A related effect size, the pooled incidence rate ratio (IRR), is instead based on counts of events over time, for example per person-year, recorded separately for each study arm. We refer to this type of data as incidence rate data.

Our interest in this problem was initiated by an analysis we recently undertook to evaluate the effectiveness of installing barriers for reducing jumping deaths at known suicide hotspots [1]. This is based on the premise that restricting access to means is one of the few successful suicide prevention strategies [2]. A total of eight studies had previously counted the number of suicide deaths at hotspots in the periods before and after the installation of barriers and safety nets. In six of these studies there were, however, no deaths following the installation of barriers.

Two approaches have been proposed for conducting meta-analyses of incidence rate data. These are the inverse-variance method [3,4] and using a Poisson regression model with fixed intervention effects [5]. The inverse-variance method is problematic when there are “structural zeros” (data like ours where multiple studies have counts of zero in one or both arms of a study) because when a study contains a zero count, the study-specific log IRR and the variance of the study-specific log IRR are both undefined. Thus all studies with zero counts are omitted from the analysis. One proposed remedy is to apply a continuity correction [6]; although this has generally only been considered when a single study has a zero count, not multiple studies. Using a Poisson regression model with *fixed* intervention effects has been proposed as a means of dealing with varying exposure time [5], but may also be useful for addressing problems where there is a number of zero counts in the data. Another option, which has not been applied to meta-analysis problems before, is to extend the previous model by estimating a Poisson regression models with *random* intervention effects instead. The advantage of this approach is that, in addition to potentially dealing with zero counts and varying exposure time, it may resolve problems that occur when there is heterogeneity in the intervention effect. This method, although widely used, has not previously been applied to meta-analysis problems.

### Study aims

The purpose of this study is to evaluate the usefulness of a Poisson regression model with random intervention effects for meta-analysis when the data contains structural zeros. We explore this using frequentist and Bayesian implementations. We make comparisons with the inverse-variance method (with and without the continuity correction) and the Poisson regression model with fixed intervention effects. This extends previous work which has focused on rare events and varying exposure time [3-5,7], but not situations that result in structural zeros or heterogeneity in the intervention effect. We evaluate these methods through Monte Carlo simulation, manipulating the number of zero counts, the amount of heterogeneity in the control and intervention groups and the number of studies within each meta-analysis. We then apply each of these methods to data from two published studies: the suicide prevention work outlined above [1], and a Cochrane review which evaluated the effectiveness of condom use in reducing heterosexual HIV transmission [8].

## Methods

### Inverse-variance methods

The inverse-variance method of meta-analysis synthesises information from multiple sources by calculating a pooled estimate of the effect size of an intervention by taking a weighted average of point estimates from independent studies. This method is widely used and recommended in the Cochrane Handbook [6] as well as other sources [3,4] for pooling incidence rate data.

When the original effect sizes are odds ratios, hazard ratios or risk ratios, then the estimates are first transformed onto the log scale, since the sampling distribution of the pooled estimate will be more approximately normally distributed than on the untransformed scale (thus improving the accuracy of inferences based on asymptotic theory) and because there is no closed form formula for the variance of these effect sizes on the untransformed scale. Risk-differences, or other absolute measures, are left on their original scale. We briefly review the formalities of the inverse-variance approach as it applies to estimating pooled IRRs and the assumptions this requires.

We are interested in making inferences about the parameter *θ*_*i*_= log(IRR_*i*_)= log(*λ*_*Ii*_/*λ*_*Ci*_), the log IRR for the *i*^th^ study (i.e. pertaining to the target population of the *i*^th^ study) where *λ*_*Ii*_ and *λ*_*Ci*_ are the event rates in the intervention and control arms respectively of the *i*^th^ study. For an individual study recording data on the counts of events over time in intervention and control groups, an estimate ${\hat {\theta }}_{i}$ of *θ*_*i*_ can be calculated from the observed log IRR as

(1) $$ {\hat{\theta}}_{i} = \log \left(\frac{{E}_{Ii} / {T}_{Ii}}{{E}_{Ci} / {T}_{Ci}}\right)  $$

where *E*_*Ii*_ and *E*_*Ci*_ represent the counts of the number of events, and *T*_*Ii*_ and *T*_*Ci*_ the exposure time (e.g. in person years), in the intervention and control groups respectively for the *i*^th^ study. Where a study contains a zero count in either the control group or the intervention group, the usual procedure is to add a “continuity correction” of 0.5 to the counts from both groups of the study [6] although other values have been proposed in the context of pooling odds ratios [9-11]. All estimates are then based on the revised group sizes. The approximate standard error of this estimate is

(2) $$ \text{SE}({\hat{\theta}}_{i}) = \left(\frac{1}{{E}_{Ii}} + \frac{1}{{E}_{Ci}}\right)^{1/2}.  $$

Under the assumption of homogeneity, namely that *θ*_*i*_ is constant across studies, a reasonable procedure to estimate the study-independent log IRR *θ* is to take an average of the study-specific estimates ${\hat {\theta }}_{i}$ weighted by the inverse of their sampling variances. The resulting pooled estimator [3,6] and its standard error are

(3) $$\begin{array}{*{20}l} \hat{\theta} & = \frac{\sum {w}_{i} {\hat{\theta}}_{i}}{\sum {w}_{i}} \end{array} $$

(4) $$\begin{array}{*{20}l} \text{SE}(\hat{\theta}) & = \frac{1}{\sqrt{\sum {w}_{i}}} \end{array} $$

where *w*_*i*_ is defined as

(5) $$ {w}_{i} = \frac{1}{\text{SE}[{\hat{\theta}}_{i}] + {\tau}^{2}}  $$

and the summations are over the study index *i*. In this formulation, the parameter *τ*^2^ represents the between-study variance of *θ*_*i*_ to account for any heterogeneity in the effect size. The parameter *τ*^2^ can be estimated using the method of moments formula [12]. Other estimators of heterogeneity have been proposed, for instance *I*^2^ [13,14], as well as methods for constructing a confidence interval of the heterogeneity estimate [15].

Before generalising the above by progressing to a regression setting, we mention a simpler method that aggregates study-specific counts and the corresponding exposure time in each arm across studies, and then calculates the pooled incidence rate ratio using these totals. This approach, sometimes referred to as the “naive method” [16,17], estimates a pooled effect size that is the ratio of exposure time-weighted averages of study-specific rates in the intervention and control arms. The study by Weller and David-Beaty [8], to which we return in the application section, provides an example of this approach. The deficiency with this method is that in order for it to produce an unbiased estimate of an intervention effect it requires the strong assumption that the population events rates in both the control arms and intervention arms do not vary between studies. Although this assumption can be tested empirically using the data it seems unrealistic and is likely to hold only very infrequently in practice. In addition, this approach has been criticised because it fails to account for between-study heterogeneity, and in the context of network meta-analysis, breaks randomisation [16,17]. As such, we do not consider this approach further.

### Poisson regression with fixed intervention effects

Inverse-variance weighted averages of study-specific log IRRs can also be achieved by using a suitably specified Poisson regression model [4,5]. In this approach, the data are set up in a long form so that each observation represents the number of events in each arm of each study (see the Additional file 1: Appendix for an example of this data structure). The model is specified in such a way that the regression coefficients represent the intervention effect and the event rate of the control group in each study. Each study arm’s exposure time is included as an offset term, with robust standard errors (with the study as the “cluster”) used to adjust the estimated precision of the estimates for any between-study variability in the intervention effect. Specifically we model the data as

(6) $$ \begin{aligned} {y}_{ij} & = {\text{Poisson}}({\mu}_{ij}) \\\ {\mu}_{ij} & = \exp[\beta_{i} + \beta_{\text{int}} \times j + \log({\text{time}}_{ij})] \end{aligned}  $$

where *i*=1,2,…,*K* represents the study index across *K* studies contributing data to the analysis, *j* represents the intervention index (coded 0 in the control group and 1 in the intervention group), *μ*_*ij*_ is the expectation of *y*_*ij*_, *y*_*ij*_ and time_*ij*_ are the event count and exposure time respectively for the *j*^th^ group in the *i*^th^ study, *β*_*i*_ is the logarithm of the event rate in the control group of the *i*^th^ study, and *β*_int_ is the logarithm of the pooled IRR.

The Poisson regression model presented above is able to accommodate baseline variability (variance in the control event rates across studies). It does, however, assume that the IRRs are constant across studies, so it does not as specified above allow for heterogeneity in the intervention effect that is not explained by baseline variability. One could fit a saturated model interacting indicator variables for study with the binary intervention variable, for example

$$ \begin{aligned} &\exp[\beta_{1} + \beta_{\text{int}} \times j + \beta_{1} \times \beta_{\text{int}} \times j + \dots + \beta_{k} + \beta_{k} \times \beta_{\text{int}}\\ &\qquad \times j + \log({\text{time}}_{ij})] \end{aligned} $$

However, this would produce a model that simply reproduces the data (i.e. observed and fitted values would be the same) and likely over-fitted (i.e. have poor predictive performance in out-of-sample testing). In the next section we consider extending the Poisson regression model to relax the assumption that *β*_int_ is constant across studies (i.e. the effect size is homogeneous across studies) and to allow for (and quantify) any heterogeneity in a parsimonious way.

### Poisson regression with random intervention effects

Poisson regression with fixed intervention effects can be extended to measure baseline variability and between-study heterogeneity in the intervention effect. We do this by declaring the study-specific parameters in the linear predictor to be random effects that are assumed to have been drawn from a distribution of such effects across a hypothetical population of similar studies. The parameters governing the distribution of these random effects are estimated from the data. One such model that allows us to assess both baseline variability and between-study heterogeneity in the intervention effect has random effects for both the intercept and intervention effect is

(7) $$  \begin{aligned} {y}_{ij} & = {\text{Poisson}}({\mu}_{ij}) \\ \ {\mu}_{ij} & = \exp[({\beta}_{1} + {\gamma}_{i0}) + ({\beta}_{\text{int}} + {\gamma}_{i1}) \times j + \log({\text{time}}_{ij})], \end{aligned}  $$

where *γ*_*i*0_∼*N*(0,*σ*^2^) and *γ*_*i*1_∼*N*(0,*τ*^2^). The parameters *β*_1_ and *β*_int_ are fixed effect regression coefficients, whereas *γ*_*i*0_ represents the study-specific deviation from the average event rate and *γ*_*i*1_ represents the study-specific deviations from the average intervention effect. Their variances, *σ*^2^ and *τ*^2^, estimate the baseline variability and between-study heterogeneity in the control group and intervention effect respectively. These are referred to as random effects variance parameters.

As with the fixed effect model, exp(*β*_int_) represents the pooled IRR. It appears in this model, however, with the explicit acknowledgement that it is a population-averaged parameter and that specific instances of the IRR may vary between the sub-populations that are the targets of each study. Note that while we focus on a two-group comparison here, this approach is very flexible and can accommodate designs where there are multiple treatment arms (i.e. a three-group comparison) through the use of indicator variables and random-effects parameters for each treatment arm in the model. We refer to this approach as “Poisson regression with random intervention effects”.

Mixed-effect models can also be fitted by taking a Bayesian approach, that is, specifying a full probability model with distributional assumptions for both the observed data and the model parameters. Such a specification is particularly suited to hierarchical models like those used in meta-analysis, where the distribution of the data (in the case of meta-analyses, the event counts) are governed by parameters (the study-specific event rates and intervention effect) that themselves have a population distribution defined by a set of hyper-parameters (the random effects variance parameters) and so on as we progress through the levels of the hierarchy. The advantage of a full probability model specification is that it produces a joint posterior probability distribution for the parameters, which allows for more flexible approach to inference and incorporates explicitly the uncertainty of estimation in all parameters. We refer to this approach as “Bayesian Poisson regression with random intervention effects”.

The Bayesian approach comes at a cost, however, and that is the need to specify prior probability distributions for the unknown parameters (a sampling distribution for the data is also required, but this is usually implicit in the proposed regression model). It is well known that inferences about variance parameters when the data are sparse can be especially sensitive to the choice of prior distributions [18-20]. In this situation, non-informative prior distributions (prior distributions that are intended to allow Bayesian inference when not much is known beyond what is available in the data) are often employed. Several non-informative prior distributions have been proposed for estimating the variance parameters for continuous (normal) outcomes, i.e. estimating $\sigma _{\alpha }^{2}$ when $\theta _{\textit {ij}} \sim \text {Normal}(\mu + \alpha _{j}, {\sigma _{y}^{2}})$ where $\alpha _{j} \sim \text {Normal}(0, \sigma _{\alpha }^{2})$. These are the inverse-Gamma distribution, the log-normal distribution and the half-Cauchy distribution [19,21,22].

When the inverse-gamma distribution is used as a prior distribution for the variance parameters, *σ*^2^∼1/*z* and *τ*^2^∼1/*z* where *z*=Gamma(*ε*,*ε*). When *ε*=0.001 this is a proper prior distribution (i.e. it does not depend on the data and integrates to 1 [23]) and close to uniform on log(*σ*) and log(*τ*). The inverse-gamma(0.001, 0.001) distribution has a peak close to zero and a long tail, meaning that low values for the variance components are supported (although when *σ*^2^ or *τ*^2^ are not close to zero this may unreasonably influence the posterior distribution).

If a log-normal prior distribution is employed as a prior distribution for the variance parameters, then the log standard deviations are normally distributed (e.g. *σ*∼Normal[0,100^2^] and *τ*∼Normal[0,100^2^]). A related prior, which allows estimation of the standard deviations on their natural scale is the half-Cauchy prior distribution, i.e. *σ*∼half-Cauchy(*C*) and *τ*∼half-Cauchy(*C*). The parameter *C* is the population median standard deviation. In a pure Bayesian analysis, the value of *C* would be based on prior information.

A final strategy is to take an empirical Bayes approach; for example, allowing the specification of the prior distribution for the variance parameters to depend on estimates of their magnitude and precision based on results from a non-Bayesian (frequentist) analysis. A possible prior distribution has the form $\sigma \sim \text {Uniform}[0, \hat {\sigma } + \text {SE}(\hat {\sigma })]$ and $\tau \sim \text {Uniform}[0, \hat {\tau } + \text {SE}(\hat {\tau })]$.

## Simulation study

### Overview

We use simulation methods to evaluate the performance of six different methods of pooling IRRs. These are (1) the inverse-variance method; (2) the inverse-variance method with the continuity correction; (3) Poisson regression with fixed intervention effects; (4) Poisson regression with random intervention effects; (5) Bayesian Poisson regression with random intervention effects with a inverse-gamma prior for the variance parameters; and (6) Bayesian Poisson regression with random intervention effects with a half-Cauchy prior for the variance parameters. The two key manipulations were the number of zero counts and the level of baseline variability and heterogeneity in each meta-analysis sample. We also varied the number of studies within each meta-analysis.

### Data generation

Table 1 shows the list of parameters and their assigned values used to simulate the data. Simulated datasets were drawn from a Poisson distribution using Eq. 7. Our simulations fixed *β*_int_ at log(0.2)=−1.609, meaning that the number of events in the intervention group were 80 percent less per unit of time than in the control group. The values for time were integer values drawn from a uniform distribution. In the control groups, time ranged from two to ten years; in the intervention group it ranged from two to five years. This mimicked the pre- and post-intervention suicide studies discussed previously where typically a larger amount of pre-intervention data was available than post-intervention.

**Table 1:** **Values used in the simulation study**

	***Assigned values***
*Methods evaluated*	
Inverse-variance method	
Inverse-variance method with continuity correction	
Poisson regression model with fixed intervention effects	
Poisson regression model with random intervention effects	
Bayesian Poisson regression model with random intervention effects using inverse-gamma priors for *τ* ^2^	
Bayesian Poisson regression model with random intervention effects using half-Cauchy priors for *τ*	
*Fixed parameters*	
Incidence rate ratio, exp(*β*_int_)	0.2
Time, intervention group, *t*_1_	*t*_1_∼Uniform(2,5)
Time, control group, *t*_0_	*t*_0_∼Uniform(2,10)
Number of simulated datasets per scenario, *B*_*s*_	500
*Varied parameters*	
Percent of zero counts in the intervention group, Poisson(*β*_1_∗0.2)	0.09%, 5%, 14%, 37%, 55%, 82%
Heterogeneity: control and intervention groups, *σ*,*τ*	
Scenario A	(0.1,0.5)
Scenario B	(0.1,2.5)
Scenario C	(1.0,0.5)
Scenario D	(1.0,2.5)
Scenario E	(0.1×*β*_1_,2.5)
Number of studies, *k*	5, 10, 20

We varied the percentage of zeros in the intervention group from approximately 0.09 percent zeros to 82 percent zeros. We did this through setting the values of *β*_1_ to log(35), log(15), log(10), log(5), log(3) and log(1) so that when we drew random observations a Poisson distribution with mean *μ*= exp(*β*_1_)×0.2, the probability *y*=0 would be 0.1, 5, 14, 37, 55 and 82 percent, respectively (ignoring random-effects). These values provided a range in which to explore the effect of increasing the percentage of zeros in the data.

We also varied the amount of baseline variability and between-study heterogeneity. We considered scenarios where baseline variability was either *σ*=0.1 or 1.0 or where the baseline variability was proportionate to the baseline event rate (*σ*=0.1× exp(*β*_1_)). Similarly, we examined two values of between-study heterogeneity in the intervention effect, *τ*=0.5 or 2.5. In all, we examined five combinations of *σ* and *τ* representing the broad spectrum in which baseline variability and heterogeneity may influence real-world data. We refer to these as “Scenario A”, “Scenario B”, and so on.

Finally, we varied the number of studies that comprised each meta-analysis, setting *k*= 5, 10 and 20. We primarily focus on reporting the results of *k*=5 and *k*=10 studies, given that this represents the typical size of a systematic review in medicine [9,24].

### Implementation

Varying six values of *β*_1_, the five heterogeneity conditions and the three study sizes, produced *s*=90 scenarios for comparison. We simulated *B*_*s*_=500 datasets for each of the 90 scenarios, giving a total of 45,000 simulations. (We chose this number of simulations to keep the computation time manageable.) We used Stata 13.1 [25] to generate the data and estimate parameters for the first four methods of interest. We estimated parameters from the inverse-variance method using the metan package [26] in Stata. We used the poisson command in Stata to estimate a Poisson regression model with fixed intervention effects and the meqrpoisson to estimate the Poisson regression model with random intervention effects. The Bayesian Poisson regression models with random intervention effects were estimated using JAGS 3.10 [27] and rjags in R 3.1.1 [28]. For the Bayesian Poisson regression model with an inverse-gamma prior distribution for the variance parameters we used *ε*=0.001. For the equivalent model with a half-Cauchy prior distribution we used *C*=1. The Bayesian models were fit using two chains with an initial burn-in of 1000 iterations, followed by sampling of 5000 iterations. We checked a random sample of simulations from each scenario to determine if the chains had mixed together and encountered no problems.

### Data extraction and analysis

From each simulation we extracted the estimated log IRR, $\hat {\beta }_{\text {int}i}$, its standard error, $\text {SE}(\hat {\beta }_{\text {int}i})$, and the estimates of the variance parameters, $\hat {\sigma }_{i}$ and $\hat {\tau }_{i}$. For $\hat {\beta }_{\text {int}i}$, we evaluate bias, accuracy and coverage for each of the 90 scenarios of interest; for $\hat {\sigma }_{i}$ and $\hat {\tau }_{i}$ we evaluate bias only [29].

Bias in the log IRR was estimated by the percentage bias $(\bar {\hat {\beta }}_{\text {int}} - \beta _{\text {int}}/\beta _{\text {int}}) \times 100$, where $\bar {\hat {\beta }}_{\text {int}} = \sum _{i = 1}^{B_{s}} \hat {\beta }_{\text {int}i} / B_{s}$ and *β*_int_= log(0.2)=−1.609. Accuracy was measured by the mean square error, $(\bar {\hat {\beta }}_{\text {int}} - \beta _{\text {int}})^{2} + (\text {SE}(\hat {\beta }_{\text {int}}))^{2}$, where $\text {SE}(\hat {\beta }_{\text {int}})$ is the empircal standard error over the *B*_*s*_ simulations. We calculated coverage, the proportion of simulation in which the 95% confidence interval $\hat {\beta }_{\text {int}i} \pm 1.96 \times \text {SE}(\hat {\beta }_{\text {int}i})$ includes *β*_int_.

The percentage bias in $\hat {\tau }_{i}$ was estimated for all models except the Poisson regression model with fixed intervention effects by $(\bar {\hat {\tau }} - \tau / \tau) \times 100$, where $\bar {\hat {\tau }} = \sum _{i = 1}^{B_{s}} \hat {\tau }_{i} / B_{s}$. Only the Poisson regression methods with random intervention effects estimate $\hat {\sigma }_{i}$. We therefore only extracted this in these cases, estimating percent bias in the same way as for $\hat {\tau }_{i}$.

## Empirical studies

### Suicides from known jumping hotspots

In the introduction we briefly described the motivating example for this simulation study – a meta-analysis of the effectiveness of installing barriers on reducing suicide by jumping at known hotspots [1]. Jumps from these sites (bridges, viaducts and cliffs) generally have high fatality rates, can cause significant distress or injury to bystanders and often receive prominent media coverage, increasing the risk of copycat acts [30]. A number of studies have investigated the effectiveness of structural interventions – such as barriers, fences or safety nets – on reducing suicide by jumping at these sites [31-39]. Individual studies are typically before-and-after designs, with the pre-intervention period considered the “control” group and the post-intervention the “intervention” group. (Although we do not show the data here, these studies also compare suicide rates at nearby sites before and after the introduction of barriers at the hotspot, thereby providing additional information on the effectiveness of barriers.)

The data from the eight studies that examined the number of suicides by jumping before and after the installation of barriers is shown in Table 2. Six of the studies had zero events after the introduction of barriers at the hotspots and exposure time ranged from approximately 5 months to 22 years. Pirkis et al. [1] reported a pooled incidence rate ratio of 0.14 with 95% CI 0.09 to 0.21, although this estimate does not include a parameter for *τ*, the random-effect parameter for between-study heterogeneity in the intervention effect. We re-analyse this data using the six methods outlined in the simulation study.

**Table 2:** **Suicide counts and exposure time by study**

	**Pre-intervention**		**Post-intervention**	
**Study no.**	**No. events**	**Time (years)**	**No. events**	**Time (years)**
1	19	6	0	4
2	41	5	20	5
3	221	14	0	0.4
4	25	7	1	5
5	14	22	0	22
6	7	3	0	3
7	96	9	0	4
8	13	10	0	2

### Condom effectiveness in reducing heterosexual HIV transmission

Weller and Davis-Beaty [8] used meta-analysis to evaluate the effectiveness of condoms in reducing the incidence of HIV infection between heterosexual couples. They included studies that examined the direction of transmission from male to female partners, female to male partners, and studies where the direction of transmission was unknown. This was based on observational data, so the “control” group was couples who never used condoms and the “intervention” group was those who always used condoms. The outcome of interest was the incidence of HIV transmission.

Weller and Davis-Beaty [8] identified 14 studies that met the inclusion criteria. The data used in their meta-analysis is shown in Table 3. The unit of analysis was the study/direction of transmission. Most studies are represented by a single row of data because they examine the transmission in one direction only; however, two studies (denoted as study numbers 1 and 2 and study numbers 11 and 12) are represented twice because they examined transmissions in two directions. We use this unit of analysis to be consistent with the original study. The *†* symbol indicates the data that Weller and Davis-Beaty used in their primary analysis.

**Table 3:** **Heterosexual HIV transmission counts and exposure time by study**

	**Always use condoms**		**Never use condoms**	
**Study no.**	**No. events**	**Person-years**	**No. events**	**Person-years**
1	0	11.5 *†*	2	6.9 *†*
2	0	8.4 *†*	4	21.1
3	1	101 *†*	13	185.3
4	1	8.54 *†*	-	-
5	-	-	1	.006
6	0	45.2 *†*	-	-
7	4	136.1 *†*	-	-
8	0	28 *†*	-	-
9	5	362.5 *†*	-	-
10	-	-	0	5 *†*
11	-	-	2	60.4 *†*
12	-	-	10	147 *†*
13	-	-	8	139.3
14	0	7.5 *†*	0	9.6
15	0	249.6 *†*	-	-
16	0	6 *†*	0	24 *†*

*†* Included in the primary analysis.

## Results

### Simulation study

**Figure 1:**
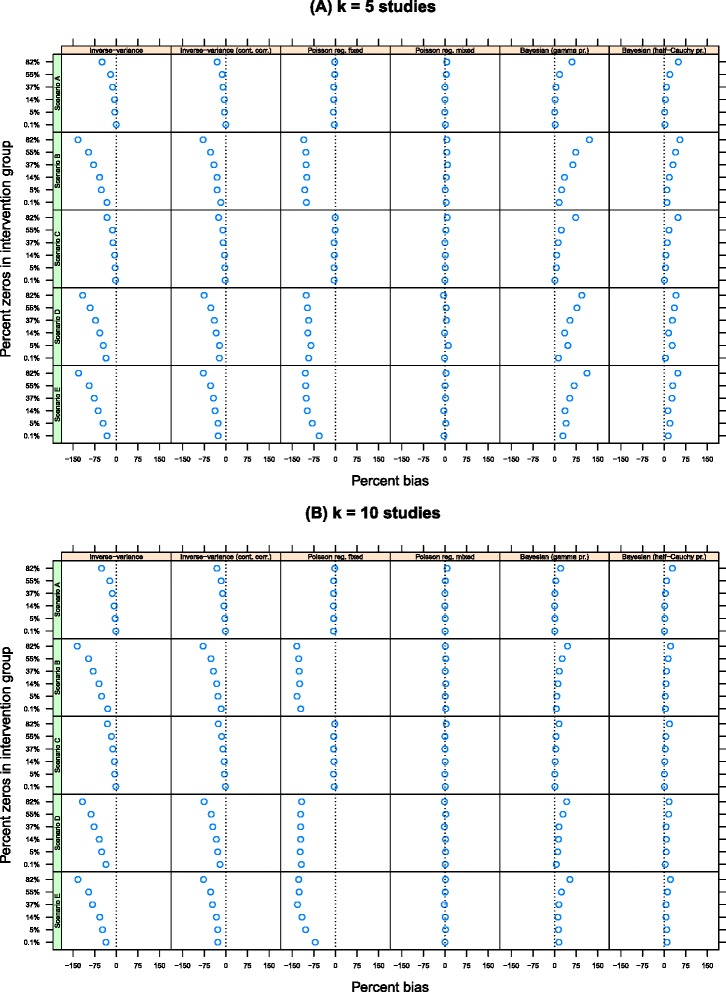
Percentage bias in the estimate of $\bar {\hat {\beta }}_{\text {int}}$ by number of studies, estimation method and percentage of zeros in the data. The true value is log(0.2)=−1.609 and the estimates are unbiased if they fall along the *x*=0 line. **(A)** k = 5 studies and **(B)** k = 10 studies.

Figure 1 shows the percentage bias in the pooled IRR on the log scale across the scenarios. The plots are grouped by method (columns) and scenario (rows) and are presented separately for *k*=5 (top panel) and *k*=10 studies (bottom panel). (All results for *k*=20 are presented in the Additional file 1: Appendix.) Within each plot, the percentage of zeros in the data increases as the values on the *y*-axis increase from approximately 0.1% zeros to 82% zeros. The pooled log IRR, $\bar {\hat {\beta }}_{\text {int}}$, is unbiased if it falls along the vertical line at *x*=0.

As the percentage of sparse data within the intervention group increases, the point estimate of the pooled log IRR derived from the inverse-variance method display increased bias (column 1). For instance, in scenario A (*σ*=0.1,*τ*=0.5) with *k*=5 studies, the percentage bias is approximately 0% when there is effectively no zeros in the data. With 5% zeros, the percentage bias is 5%, increasing to 50% when there is around 82% zeros in the data. This pattern is replicated for all other scenarios and when *k*=10 and *k*=20 (see the Table A1 in the Additional file 1: Appendix). This pattern occurs because the log IRR for an individual study is undefined when there are zero events, and as a result, studies with zeros are excluded from the estimate of the pooled effect size, biasing the results. This pattern is exacerbated by larger heterogeneity values (*τ*=2.5 in scenarios B, D and E). For instance, when there is 82% zeros in the data, the percent bias is 133%, 116% and 130% in these scenarios respectively for *k*=5, and by a similar amount for *k*=10 and 20. The continuity correction does not remedy any of these problems (column 2).

The Poisson regression model with a fixed intervention effect (column 3) displays only a small amount of bias in the pooled log IRR when there is low baseline variability (*σ*=0.1 in scenarios A and C). In these scenarios, the percentage bias ranged from 0.6% to 8% for all values of *k*. But when heterogeneity was larger (*τ*=2.5 in scenarios B, D and E) then this method produced point estimates of the pooled log IRR that diverge substantially from their true values. For example, the percentage bias in scenario B is approximately 100% for *k*=5 regardless of the amount of zeros in the data, and range from 120% to 134% when *k*=10. In contrast to this, the Poisson regression model with random intervention effects (column 4) produced estimates of the pooled log IRR that were close to their true value in all scenarios. The percentage bias ranged from approximately 0% to 11% for *k*=5 and between 0% and 7% when *k*=10. The size of any bias was unrelated to the percentage of zeros in the data or by baseline variability and between-study heterogeneity.

A Bayesian approach to the Poisson regression model with random intervention effects produced biased pooled log IRRs. When the variance parameters were estimated using an inverse-gamma prior distribution, the pooled log IRR was close to the true value in scenarios when there was only a small amount of zeros in the data (column 5). For instance, when *k*=5 and there was no zeros in the data, the percentage bias was just 2.2% in scenario A, 16% in scenario B, 6% in scenario C, 13% in scenario D and 29% in scenario E. As the percentage of zeros in the data increased, the amount of bias increased, such that, for instance, when there were 82% zeros in the data, the percentage bias was 61%, 121%, 73%, 95% and 112% in scenarios A through E respectively. While these effects were attenuated when *k*=10 and *k*=20 (Additional file 1: Appendix, Table A1), the general pattern remained. A similar picture emerged when the variance parameters were estimated using a half-Cauchy prior distribution (column 6).

**Figure 2:**
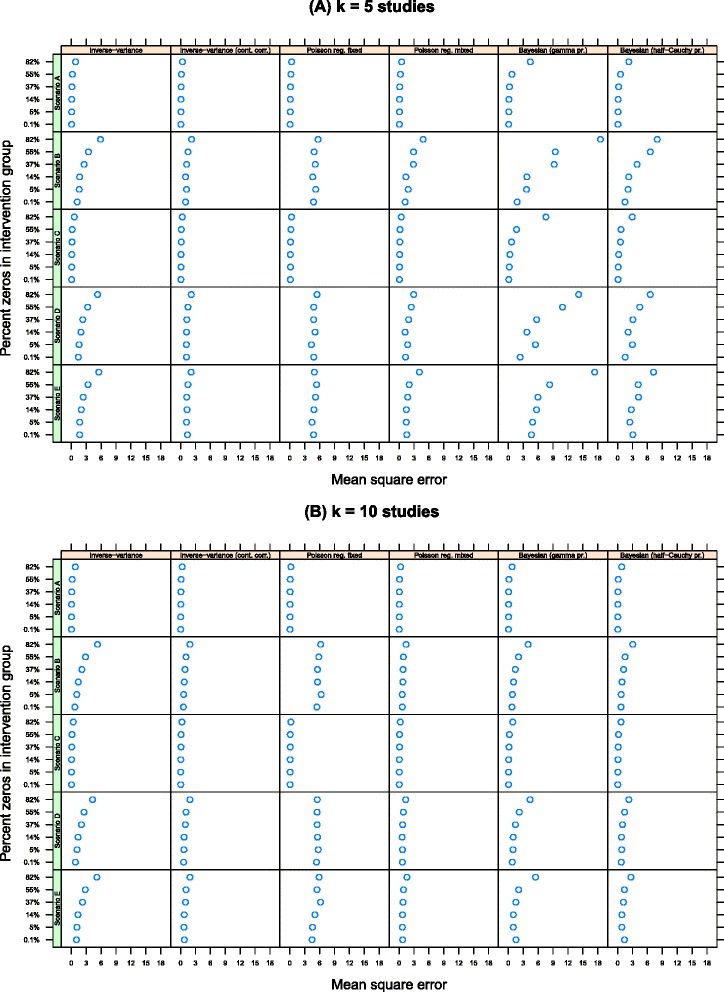
Mean square error by number of studies, estimation method and percentage of zeros in the data. Lower values are preferable to higher values. **(A)** k = 5 studies and **(B)** k = 10 studies.

Figure 2 shows the accuracy of the different methods for *k*=5 and *k*=10 measured by the mean square error. The plots are arranged as above. Smaller mean square error values are preferable to larger values, all else being equal. When *k*=5, all methods show larger mean square error values when there is a a high percentage of zeros in the data compared with when there is only a small amount of zeros. For example, for scenario A, using the inverse-variance method, mean square values range from 0.06 (when there is approximately 0.1% zeros in the data) to 0.85 (when there is 82% zeros). Mean square error values were largest on average for the *Bayesian* Poisson regression models with random intervention effects and smallest for the inverse-variance method with the continuity correction. The Poisson regression model with random intervention effects had the next smallest mean square error values on average. When *k*=10, the mean square error was much smaller for all models and the Poisson regression model with random intervention effects had the smallest average value in four of the five scenarios.

**Figure 3:**
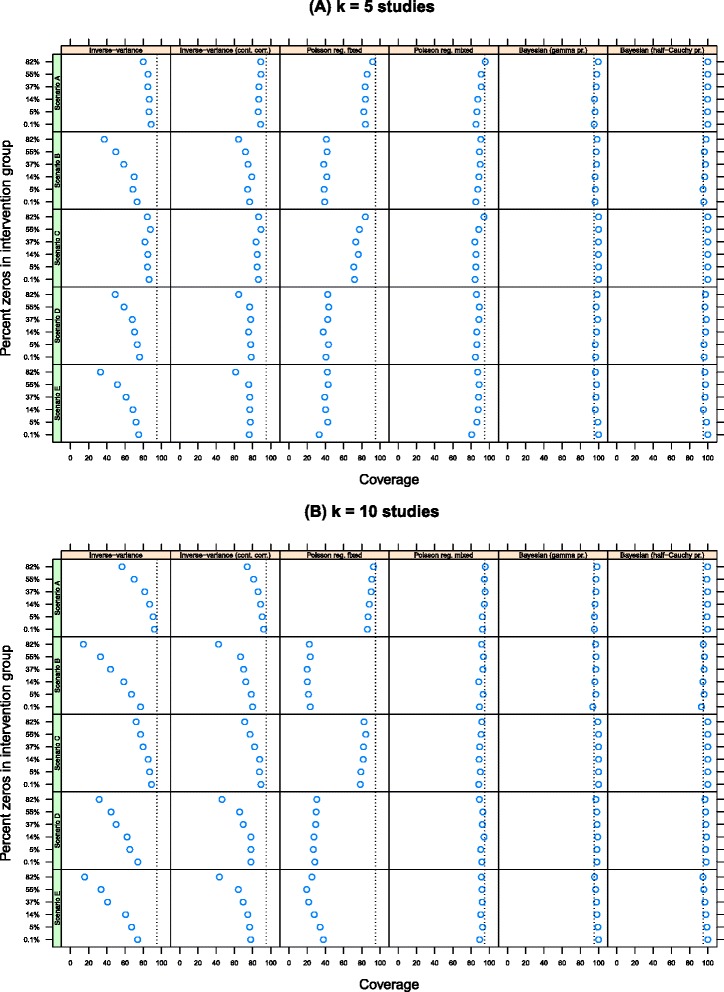
Coverage by number of studies, estimation method and percentage of zeros in the data. Methods with good coverage will have values close to *x*=95 percent. **(A)** k = 5 studies and **(B)** k = 10 studies.

Figure 3 shows the coverage of each method. The line at *x*=95 indicates the nominal 95% confidence interval (i.e. where estimated confidence interval includes the true value in 95% of simulations). The inverse-variance methods (with and without the continuity correction) and Poisson regression model with fixed intervention effects (columns 1–3) had consistently poor coverage for both values of *k*. The coverage for the Poisson regression model with random intervention effects was slightly below the nominal 95% value while the coverage for the two Bayesian implementations of this model was slightly above the 95% value.

**Figure 4:**
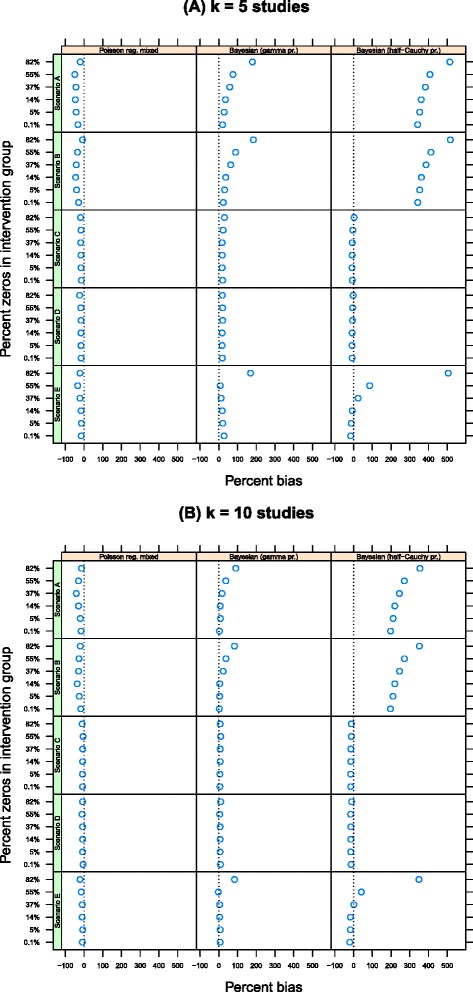
Percentage bias in the estimate of $\bar {\hat {\sigma }}$ by number of studies, estimation method and percentage of zeros in the data. The estimates are unbiased if they fall along the *x*=0 line. **(A)** k = 5 studies and **(B)** k = 10 studies.

**Figure 5:**
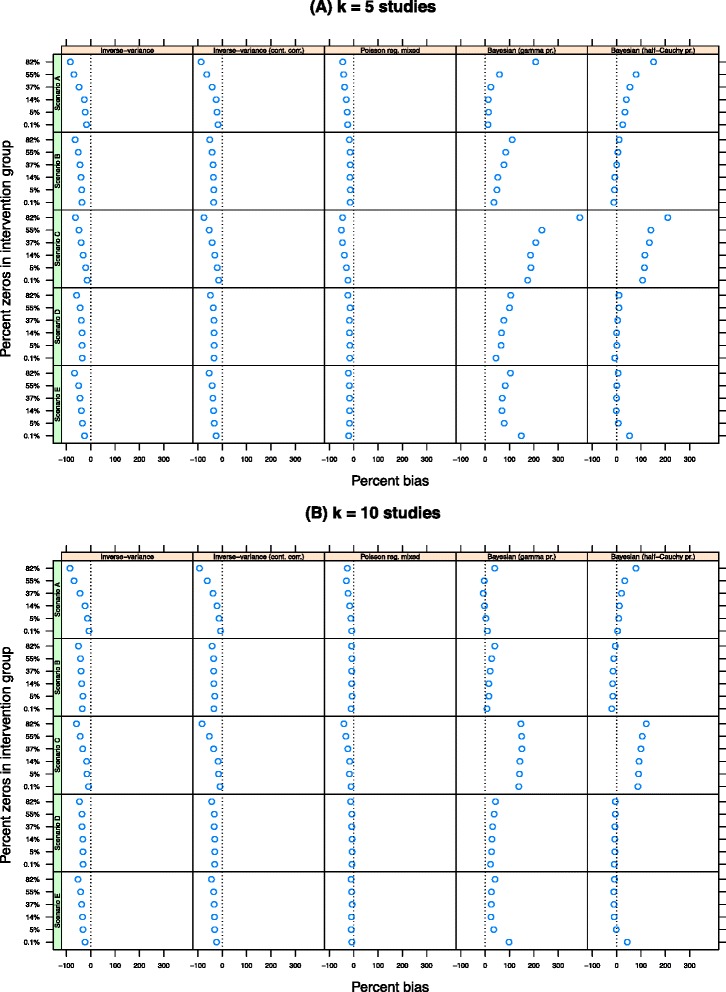
Percentage bias in the estimate of $\bar {\hat {\tau }}$ by number of studies, estimation method and percentage of zeros in the data. The estimates are unbiased if they fall along the *x*=0 line. **(A)** k =n 5 studies and **(B)** k = 10 studies.

Finally Figures 4 and 5 show the percentage bias in the variance components $\hat {\sigma }$ and $\bar {\hat {\tau }}$ by method (noting that $\hat {\sigma }_{i}$ is not estimated using inverse-variance methods and that neither $\hat {\sigma }_{i}$ nor $\hat {\tau }_{i}$ is estimated with the Poisson regression model with fixed intervention effects). For $\bar {\hat {\sigma }}$, all methods displayed bias, but the bias was smallest and most consistent for the the Poisson regression model with random intervention effects (range 20% to 49% bias for *k*=5 in scenario A versus, for instance, 20% to 180% for the equivalent Bayesian Poisson regression model with a gamma prior distribution for the variance components.) A similar picture emerged for $\bar {\hat {\tau }}$. All methods displayed bias and the bias was exacerbated by the percentage of zeros in the data and the number of studies within each meta-analysis. But the bias for the Poisson regression model with random intervention effects was generally smaller than for the competing methods, especially when *k*=10 or 20 (Additional file 1: Appendix, Tables A4 and A5). For example, in scenario A when *k*=10, the percentage bias ranged from 7% to 85% using the inverse-variance method but from 8% to 26% using the the Poisson regression model with random intervention effects.

### Empirical studies

Table 2 shows the counts of jumping suicides and the exposure time for each study reported by Pirkis et al. [1]. The zero values in six of the intervention groups means the study-specific log IRR and its standard error can only be calculated for the two remaining studies (studies 2 and 4). Therefore, analysis using the inverse-variance method estimated a pooled IRR of 0.207 with 95% confidence interval (CI) 0.026 to 1.646. This finding can be interpreted as providing insufficient evidence to conclude whether or not the barriers reduce the number of suicide jumping deaths per year. Repeating the analysis using the continuity correction meant that all eight studies could be included in the analysis and this approach yielded a pooled IRR of 0.085 with 95% CI 0.026 to 0.284, suggesting strong evidence of a protective effect. Analysis using a Poisson regression model with fixed intervention effects estimated a pooled IRR of 0.151 with 95% CI 0.089 to 0.229 and a Poisson regression model with random intervention effects estimated a pooled IRR of 0.008 with 95% CI 0.0002 to 0.300. The estimates of the random effects parameters varied between methods. Using inverse-variance methods, $\hat {\tau } = 1.34$ and with the continuity correction $\hat {\tau } = 1.25$. In a a Poisson regression model with random intervention effects, $\hat {\tau } = 2.48$. The results using the two Bayesian approaches gave a similar effect size for the pooled IRR and the estimate of heterogeneity.

Table 3 shows the counts of HIV infections and exposure time in 11 studies that followed heterosexual couples who “always” used condoms (the intervention group) and 10 studies of couples who “never” used condoms (the control group). The data pose a number challenges for traditional meta-analysis. Only 5 studies have data in both treatment arms, and unusually, there is less data available for the control group than the intervention group. The data were also sparse, with seven studies in the intervention and three studies in the control group having zero counts. The combination of these two elements means that the study-specific log IRR and its standard error were undefined for all studies. Therefore, it is not possible to calculate a pooled IRR using the inverse-variance method. Weller and Davis-Beaty [8] overcame this problem by collapsing all the data into a single table and calculating a pooled IRR from this aggregated information. This approach estimated a pooled IRR of 0.198. Weller and Davis-Beaty derive their confidence limits using a best case/worst case scenario. But using an aggregated analysis gives a 95% CI of 0.081 to 0.470.

Estimates from a Poisson regression model with fixed intervention effects could not be estimated reliably (due to the imbalance in the number of studies in the control and intervention groups), but they could be derived when the indicator variables were omitted. This gave a pooled IRR of 0.198 (95% CI 0.090 to 0.437) which is very similar to the estimates from the aggregated analysis. Analysis of this data using a Poisson regression model with random intervention effects estimated a pooled IRR of 0.171 with 95% CI 0.057 to 0.515. Weller and Davis-Beaty [8] excluded a number of studies from their primary analysis because of concerns about heterogeneity. Our simulation results suggested that the intervention effect parameters are unbiased in the presence of heterogeneity when using a Poisson regression model with random intervention effects. Therefore, we re-analysed their data using all the available information (this information also contained in Table 3). The revised analysis estimated a pooled IRR of 0.147 (95% CI 0.053 to 0.407). Using a Bayesian Poisson regression approach with an inverse-gamma distribution for the variance components gave an IRR of 0.122 with 95% credible interval 0.014 to 0.396. Using a half-Cauchy prior distribution for the variance components yielded a pooled IRR of 0.102 with 95% credible interval 0.010 to 0.500. Turning to the random effects, the Poisson regression model with random intervention effects gave an estimate of $\hat {\tau } = 0.616$. Using the inverse-gamma prior gave an estimate of $\hat {\tau } = 0.666$ while the half-Cauchy prior gave $\hat {\tau } = 0.841$.

## Discussion

Methods for the meta-analysis of incidence rate data (counts of events in time) have received relatively little attention [40], and no work has addressed how to undertake a meta-analysis when there are structural zeros in the data (multiple studies within a meta-analysis which have counts of zero events). Nonetheless, there is a need to undertake meta-analyses on this type of data. We have shown that the inverse-variance method of meta-analysis (one of the most commonly used and recommended methods) is biased in the presence of structural zeros. We show that this finding holds even after adjustment using the continuity correction as recommended by the Cochrane Handbook [6].

We explored several alternatives to the inverse method. In the context of pooling rates when exposure time varies between groups, Guevara et al. [5] proposed using Poisson regression with indicator variables for each study. Since the Poisson distribution includes zeros [41,42], this suggests a potentially useful means of pooling incidence rate data with structural zeros. The issue that then arises is how the pooled IRR and the other fixed-effects estimates are effected by baseline variability and heterogeneity. Theoretically, this approach accounts for baseline variability (i.e. variability in the incidence rates in the control group) via the use of indicator variables for each study. Our simulations show that this method produces relatively unbiased estimates when there is low baseline variability. But as the level of baseline variability increases the method displays bias in the pooled IRR. Interestingly, this bias is constant across simulations regardless of the amount of zeros in the data. Our simulations also show that Poisson regression with fixed intervention effects has high mean square error (relative to the competing method) under high heterogeneity conditions and has poor coverage. As such, we do not recommend using Poisson regression with fixed intervention effects for meta-analysis unless the baseline variability and between-study heterogeneity are both close to zero.

Our primary interest was in comparing the two aforementioned methods to Poisson regression with random intervention effects. This method extends Poisson regression with fixed intervention effects by allowing study-specific random intercepts and slopes. These parameters therefore estimate the baseline variability and between-study heterogeneity in the intervention effect. This latter estimate is often of interest when conducting meta-analysis. We explored several implementations of this method: one based on adaptive Gaussian quadrature estimation (referred to as a frequentist model), and two based on Bayesian techniques (a full probability model with prior distributions for all parameters including the study-specific intercepts and slopes). We tested the usefulness of an inverse-gamma prior distribution and the half-Cauchy distribution for the random-effect parameters while using the traditional non-informative normal distribution for the fixed-effect parameters.

Our simulations show that the (frequentist) Poisson regression model with random intervention effects estimated the pooled IRR without bias, generally had the lowest mean square error and had good coverage. These results held in a variety of situations, for instance when there was only a small number of studies in each meta-analysis, when there was high baseline variability or high heterogeneity, and when there was a large number of zeros in the data. The estimates of baseline variability and between-study heterogeneity were close to their true values, but did exhibit bias in some circumstances – most notability when there was a small number of studies in each meta-analysis and when there was a large number of zeros in the data. It is worth pointing out, however, that all methods did poorly in these situations, and that Poisson regression with random intervention effects had the lowest bias of those tested. Neither Bayesian implementation of this method were able to estimate the pooled IRR or the variance components as accurately. Based on these findings, we see Poisson regression with random intervention effects as a useful method for conducting a meta-analysis of incidence rate data, especially when the data contains structural zeros. In line with this, we give code in the Additional file 1: Appendix for setting up the data and undertaking analysis using Stata [25]. There are two important caveats to this recommendation. First, our simulations show that the accuracy of the pooled IRR improves as the number of studies increases. Thus, while it is possible to conduct a meta-analysis using, for example five studies, a meta-analysis with more studies than this will provide more stable estimates for all parameters. Second, our results show that the estimates of baseline variability and between-study heterogeneity remain biased regardless of the number of studies in the meta-analysis. As such, while the pooled IRR is likely to be accurate, the variance parameters will be estimated with error.

Although not reported here, our simulations included several other methods. We evaluated two other fixed-effects methods – complete pooling of the data to calculate the pooled incidence rate ratio and stratified pooling (by study) to calculate the pooled effect size [43]. In simulations, these results were effectively the same as those for the fixed-effects Poisson regression. We also explored several other prior distributions for the variance components in a Bayesian analysis – a log normal prior distribution for the variance components and an Empirical Bayes approach. Results for both were similar to that reported for the Bayesian methods reported here. In general, we found that the Bayesian approach was able to reproduce the results from adaptive Gaussian quadrature but we believe its performance could be improved by taking an iterative approach to determine the parameters defining the prior distribution of the variance components – an approach that has been demonstrated previously [44]. Finally, it is worth noting, that the BUGs language, the tool used to implement Bayesian analysis, is very flexible and able to draw from a complex structure for the random-effects parameters. Thus, there is likely to be situations where a Bayesian approach will out-perform a frequentist analysis.

Our study has several limitations. First, we evaluated only six methods of analysing incidence rate data, but a number of different methods have been proposed, for example, the Mantel Haenszel method [45], Peto’s method [46], the Binomial-Normal method [7]. It may be fruitful for future research to compare and contrast these methods with our preferred approach. Second, our simulations did not allow for a correlation between baseline variability and heterogeneity. This was mainly because with *k*=5 or 10 studies, there is likely to be insufficient information in the data to estimate this parameter reliability. Nonetheless, in real-world data, such an association could plausibly occur. Finally, we did not directly manipulate the sample sizes in each study. Yet, in typical meta-analyses, for example where the effect size of interest is a pooled odds ratio or rate ratio, then studies with large numbers will tend to have a strong influence on the overall result. This effect of this on estimating a pooled IRR using Poisson regression with random intervention effects is unknown.

## Conclusion

Our approach is a simple yet flexible method of undertaking meta-analyses on incidence rate data when there are zero counts in the data. Our proposed method of using Poisson regression with random intervention effects has several merits. First, many popular statistical programs (e.g. Stata, R) can perform the analysis using routinely available command. In Stata, the command is meqrpoisson and in R, the glmer command in the lme4 package. We give example Stata code in the Additional file 1: Appendix. The commands also enable the basic model to be extended – for instance it is trivial to estimate a correlation between *σ* and *τ* with modern statistical software. This is also true of Bayesian methods as implemented by JAGS, WinBUGS and OpenBUGS. Second, because the method is based on regression techniques, in principle it is possible that the models themselves can be extended to include additional covariates. For example, it is common to report separate meta-analyses for subgroups such as males and females, or for observational studies and randomised control trials. When data is available at the subgroup level, parameters representing these groups could be entered into the model either as additive terms or multiplicative terms (for instance, with the variable representing the treatment arm). Further research could investigate this more fully.

## Availability of supporting data

The methods used to generate the simulated datasets are described in the methods section. The data used in the examples is contained in Tables 2 and 3.

## Additional file

Additional file 1
**Appendix.**
