# Supplementary material for: Meta-analysis of incidence rate data in the presence of zero events
Source: BMC Med Res Methodol. 2015 Apr 30;15:42. doi: 10.1186/s12874-015-0031-0 (PMC4422043; doi:10.1186/s12874-015-0031-0)

Appendix

Stata code for fitting a Poisson regression model with random intervention effects

The data should be arranged in a long format for estimating a Poisson regression model with random intervention effects. The dataset should contain a study identifier, a variable representing the study arm, counts of the number of events in each treatment arm, and the corresponding exposure time. An example of this is shown below using the data from the suicide example. The variable `ID` is the study identifier, `tx` is the treatment arm (0 represents the pre-intervention data; 1 represents post-intervention data), `count` is the number of events and `time` is the exposure time, in this case measured in years.

```
+----------------------------+
| ID       tx   count   time |
|----------------------------|
|  1       0       25      7 |
|  1       1        1      5 |
|  2       0       19      6 |
|  2       1        0      4 |
|  3       0        7      3 |
|  3       1        0      3 |
|  4       0       41      5 |
|  4       1       20      5 |
|  5       0       14     22 |
|  5       1        0     22 |
|  6       0       13     10 |
|  6       1        0      2 |
|  7       0       96      9 |
|  7       1        0      4 |
|  8       0      221     14 |
|  8       1        0     .4 |
+----------------------------+
```

In Stata 13.1, the model is estimated using the command:

```
meqrpoisson count i.tx exposure(time) irr || ID: tx, stddev
```

Incidence rate ratios are obtained by using the option `irr`. Where the variance components are not close to zero, the model can also be fit with the command `mepoisson`.

Simulation study: Results using $k = 20$ studies for each meta-analysis

In our simulation study, we report the results of setting the number of studies comprising each meta-analysis to $k = 5$ and $k = 10$. Here we show the results of our simulations when $k = 20$. The values of all other parameters are the same as that reported in the main text.

Figure A1: PercentagebBias in the estimate of $\bar{\hat{\beta}}_{\text{int}}$ with $k = 20$ studies and by estimation method and percentage of zeros in the data. The true value is $\log(0.2) = -1.609$ and the estimates are unbiased if they fall along the $x = 0$ line.

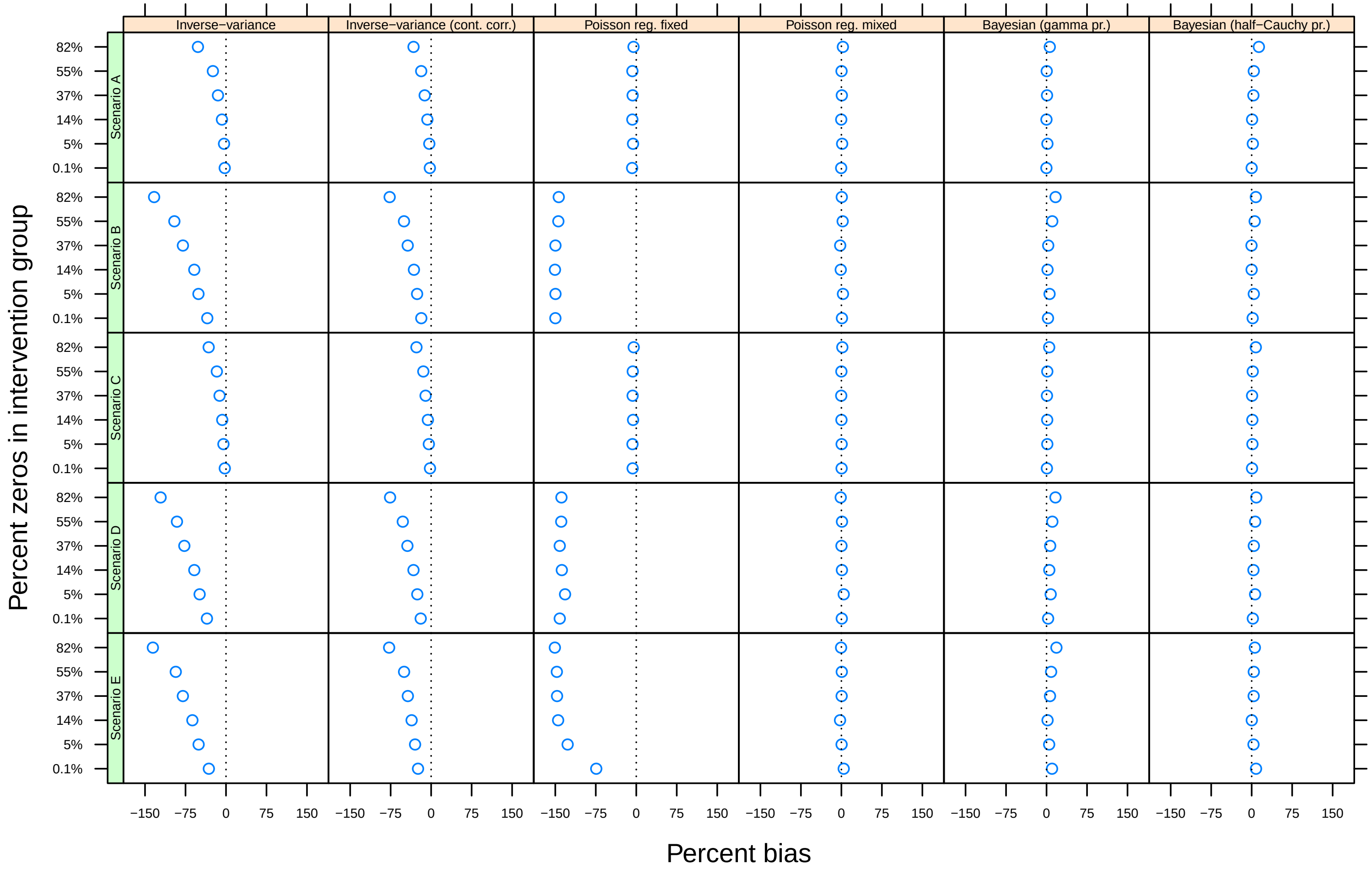

Figure A2: Mean square error with $k = 20$ studies and by estimation method and percentage of zeros in the data. Lower values are preferable to higher values.

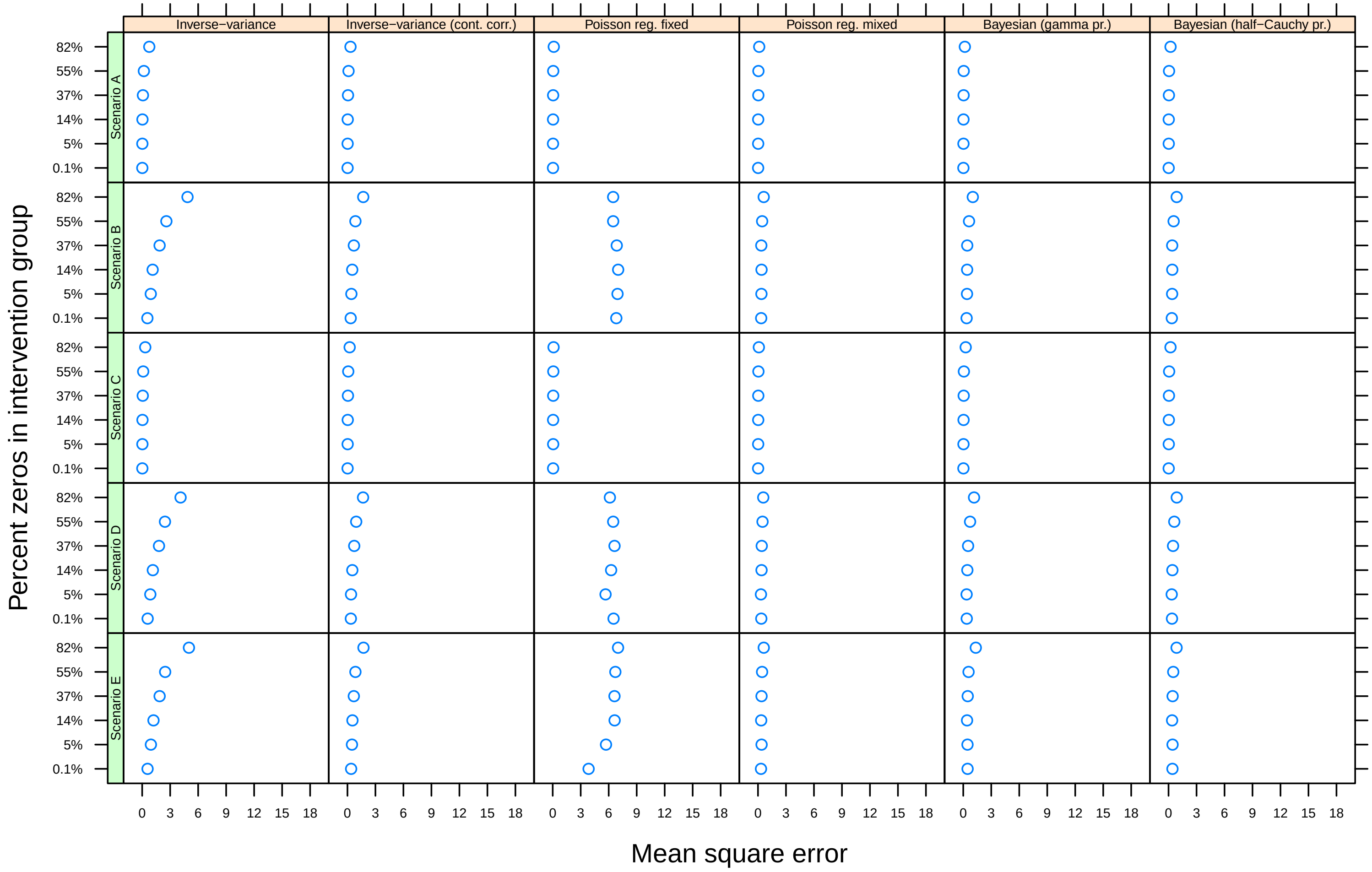

Figure A3: Coverage with $k = 20$ studies and by estimation method and percentage of zeros in the data. Methods with good coverage have values close to $x = 95$.

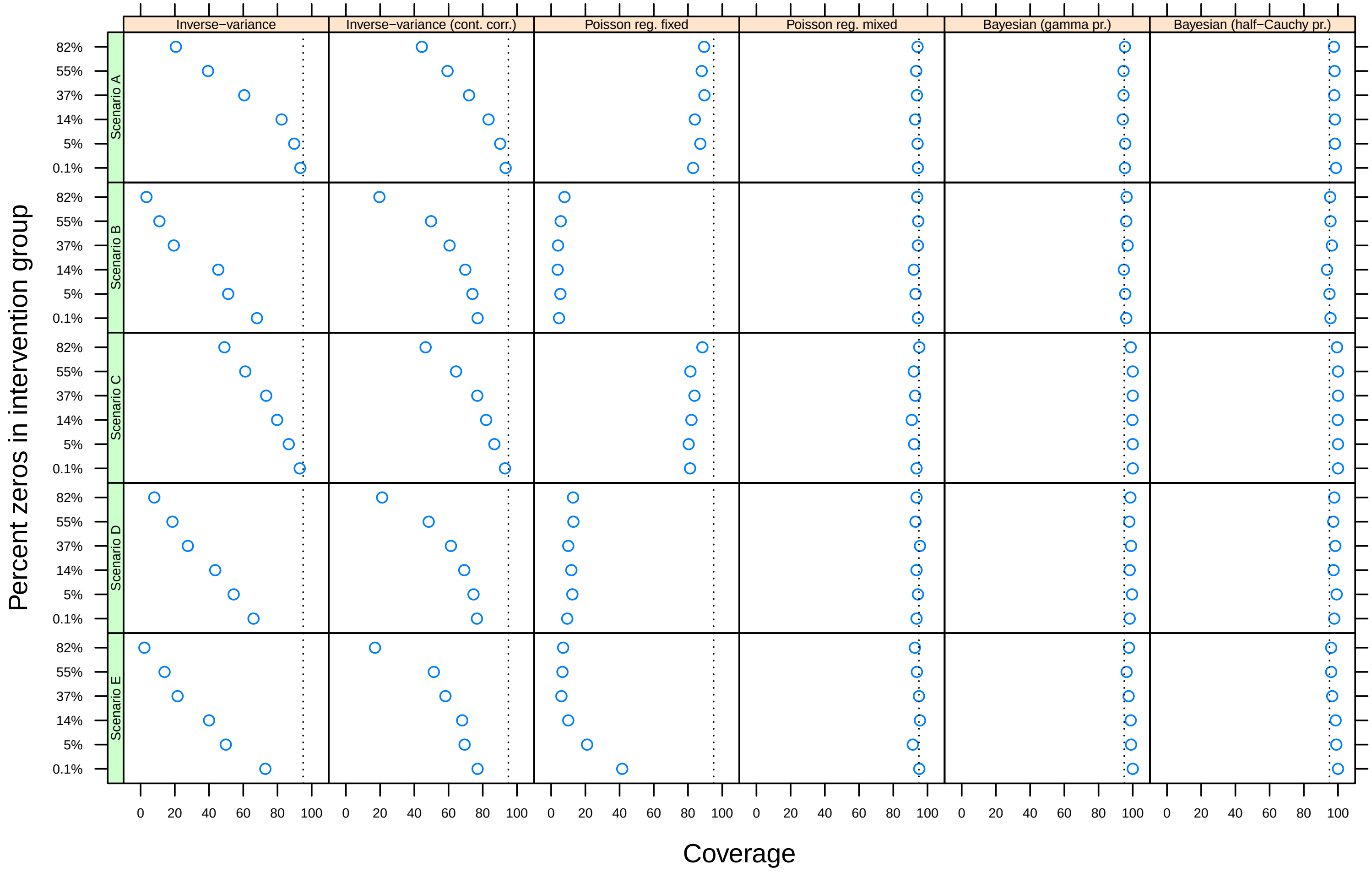

Figure A4: Percent bias in $\bar{\hat{\sigma}}$ with $k = 20$ and by estimation method and percentage of zeros in the data. The estimates are unbiased if they fall along the $x = 0$ line.

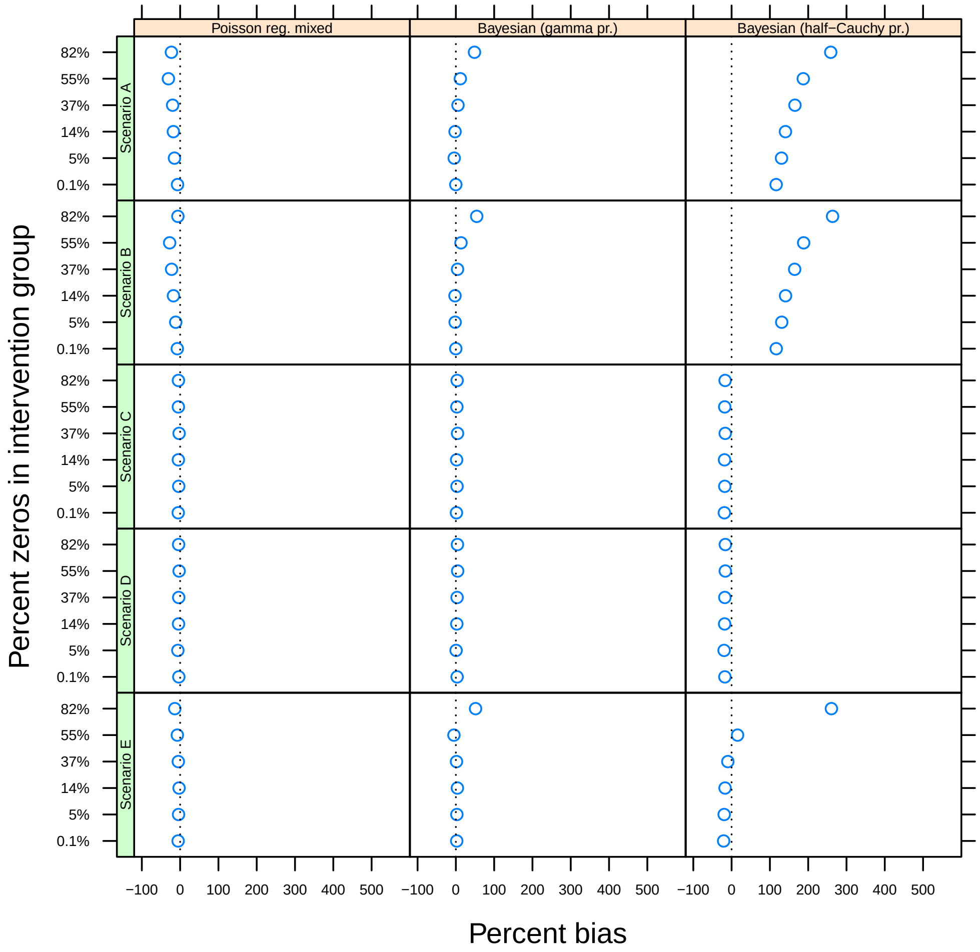

Figure A5: Percent bias in $\bar{\hat{\tau}}$ with $k = 20$ and by estimation method and percentage of zeros in the data. The estimates are unbiased if they fall along the $x = 0$ line.

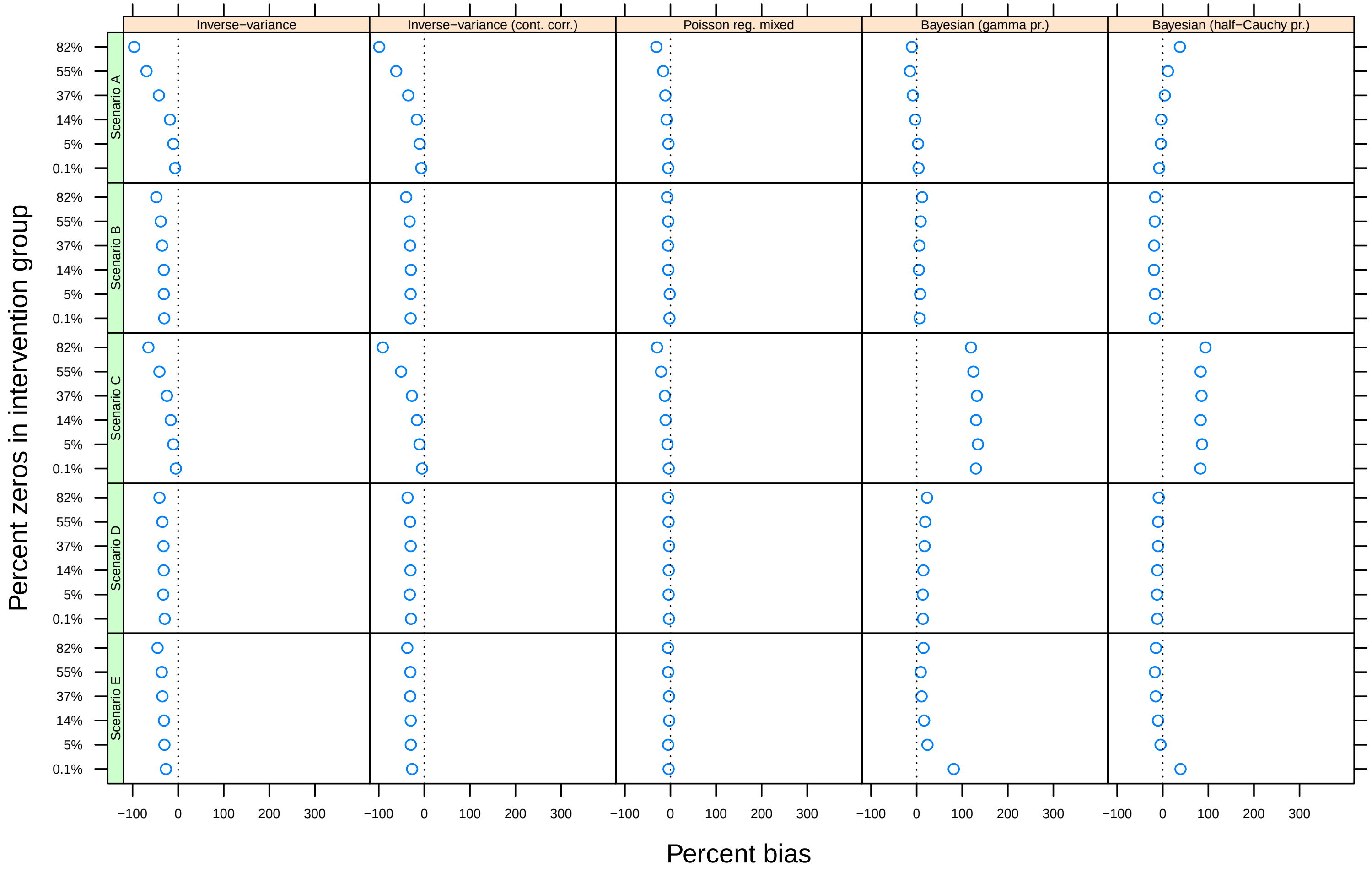

Supplement: Additional file 1 — Appendix. [file 12874_2015_31_MOESM1_ESM.pdf]
